# Clinical therapeutic effects of eradication of *Helicobacter pylori* in treating patients with type 2 diabetes mellitus: A protocol for systematic review and meta-analysis

**DOI:** 10.1097/MD.0000000000026418

**Published:** 2021-07-09

**Authors:** Zhi-Guo Shi, Li-Hui Chen

**Affiliations:** Department of Laboratory, Jincheng People's Hospital, Jincheng, Shanxi, China.

**Keywords:** efficacy, eradication, *Helicobacter pylori*, meta, type 2 diabetes mellitus

## Abstract

**Background:**

Previous studies have demonstrated that *Helicobacter pylori* is a critical factor in the development of gastrointestinal diseases. However, only limited studies have reported results on the relationship between *H pylori* infection and patients with type 2 diabetes mellitus (T2DM). Moreover, the conclusions from these past studies are variable. Because there are contradictory results on this issue, the present study aims to examine the clinical therapeutic impacts of *H pylori* eradication to treat patients experiencing T2DM.

**Methods:**

The present protocol is drafted according to the provisions of the Preferred Reporting Items for Systematic Review and Meta-analyses Protocols guidelines. PubMed, Cochrane Central Register of Controlled Trials databases, EMBASE, Web of Science, China National Knowledge Infrastructure, and Chinese BioMedical Literature Database will be searched up to May 2021 to obtain randomized controlled trials evaluating the clinical therapeutic effects of *H pylori* eradication to treat patients experiencing T2DM. We will use 2 investigators independently to carry out study selection, data extraction, and employ the Cochrane Collaboration criteria to evaluate their risks of bias. Furthermore, we will apply Stata 16.0 software to perform data analysis.

**Results:**

We intend to evaluate the clinical therapeutic impacts of *H pylori* eradication to treat patients suffering from T2DM.

**Conclusions:**

Our findings may support existing evidence on the clinical therapeutic impacts of *H pylori* eradication to treat patients with T2DM.

**Ethics and dissemination:**

Since all data will be extracted from the published literature, the study does not require an ethical approval.

**OSF registration number:**

May 31, 2021.osf.io/qtexu. (https://osf.io/qtexu/)

## 1 Introduction

Presently, diabetes is considered as one of the critical public health concerns worldwide, with an increasing prevalence. Data obtained from the International Diabetes Federation in 2017 alone points out that about 425 million people had diabetes.^[1]^ Meanwhile, data from the World Health Organization put forward that Type 2 diabetes mellitus (T2DM) is the most common type of diabetes, with an estimated 90% diabetes cases experiencing it.^[2]^ At the same time, another survey conducted in 2013 supposes that the prevalence of T2DM in China has rapidly grown.^[3]^ More specifically, diabetes severely impacts patients’ quality of life and increases the risk of mortality rates among adults in China.^[4,5]^ Particularly, the risks factors of diabetes, including genetics, adiposity, and smoking have been well established in the recent past. However, only limited studies have examined whether infectious pathogens are also responsible for pathogenesis of diabetes.

*Helicobacter pylori* is considered a gram-negative bacterium with a helical or spiral shape. It has been isolated from human stomach across different regions. An estimated 50% of the world's population can be said to have been infected with *H pylori*.^[6–8]^ Moreover, many studies have sought to understand the relationship between *H pylori* infection and diabetes patients.^[9–11]^ However, many studies have sought to understand the high prevalence of *H pylori* infection among patients with diabetes mellitus and metabolic syndrome.^[12–14]^ The present study will focus on the clinical therapeutic impacts of *Helicobacter pylori* eradication to treat patients suffering from T2DM. We believe that our study is the first comprehensive systematic review to assess the clinical therapeutic effects of *H pylori* eradication to treat patients experiencing T2DM.

## 2 Methods

The present protocol was drafted according to the provisions of the Preferred Reporting Items for Systematic Review and Meta-analyses Protocols (PRISMA-P) 2015 statement. The protocol has been registered in the Open Science Framework (DOI 10.17605/OSF.IO/QTEXU).

### 2.1 Eligibility criteria

#### 2.1.1 Types of participants

We will consider patients diagnosed with T2DM in the present study.

#### 2.1.2 Types of interventions

We will also include patients who received *H pylori* eradication treatment after the diagnosis of T2DM as the experimental group. Meanwhile, we will include patients who received other treatment or no treatment as the control group.

#### 2.1.3 Types of studies

We will rely on the randomized controlled trials to evaluate the clinical therapeutic impacts of *H pylori* eradication to treat patients experiencing T2DM. We will consider unpublished randomized controlled trials or those published as abstract, theses, or monographs to be eligible for this analysis.

#### 2.1.4 Types of outcome measures

Our main outcome of interest is the successful eradication of *H pylori.* The secondary outcomes include blood glucose control level, occurrence of adverse events, and level of patient compliance.

### 2.2 Search methods for the identification of studies

#### 2.2.1 Electronic searches

We will search PubMed, Cochrane Central Register of Controlled Trials databases, EMBASE, Web of Science, China National Knowledge Infrastructure, and Chinese BioMedical Literature Database up to May 2021 to obtain randomized controlled trials that evaluate the clinical therapeutic impacts of *H pylori* eradication to treat patients suffering from T2DM. Also, we will consider related terms, including diabetes, “diabetes mellitus,” “*Helicobacter pylori,*” “*H Pylori*,” and “*Campylobacter pylori*” to obtain data. In case of any disagreement during the electronic search process, we will address them through discussion.

#### 2.2.2 Searching other resources

We will also search ClinicalTrials.gov (https://clinicaltrials.gov/) and gray literature to establish any potential studies. Accordingly, we will review papers and bibliographies included in the studies.

### 2.3 Data collection and analysis

#### 2.3.1 Selection of studies

**Figure 1:**
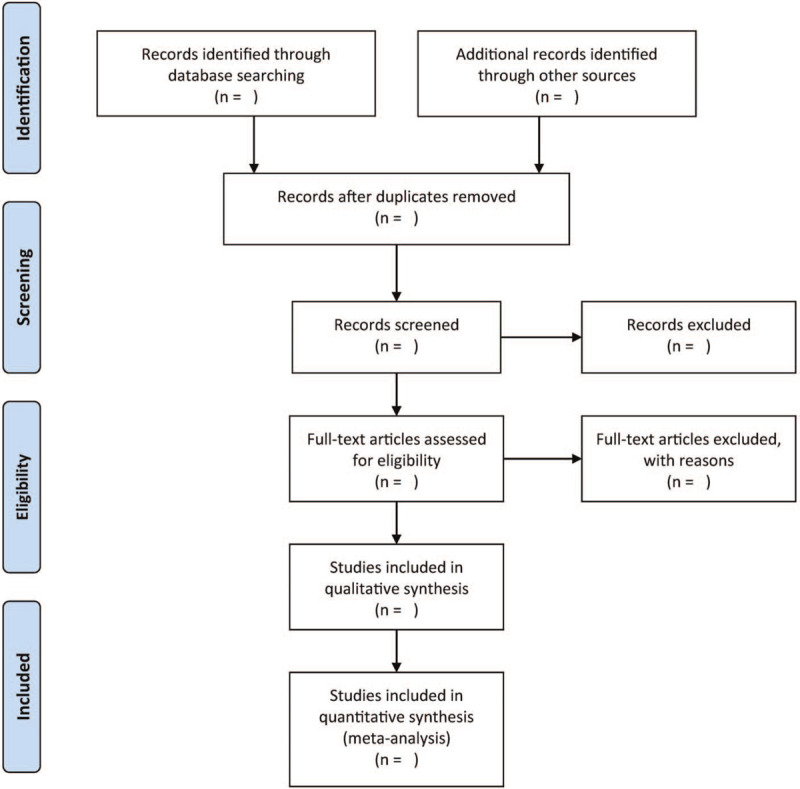
Flow diagram of study selection.

We will rely on 2 independent authors to screen and select eligible studies. We also intend to screen titles/abstracts so as to exclude irrelevant studies. After that, we will obtain full-text articles of relevant studies. We will then read these pertinent studies to assess their eligibility for inclusion. Then, we will detail reasons for excluding other studies. In case of an agreement during this process, we will address it through discussion. Figure 1 illustrates the eligibility screening process.

#### 2.3.2 Data extraction and management

We will use 2 independent authors to extract the study data. We will then use a structured checklist to extract author's name, date of publication of the article, country, ethnicity, study subjects, mean age, gender, sample size, type of diabetes, outcome measures, and method of bacteria detection. In case of an agreement during this process, we will address it through discussion.

#### 2.3.3 Assessment of risk of bias for included studies

The study will also rely on the use of the revised Cochrane risk of bias tool to assess the risk of bias for the studies selected.^[15]^ The potential risks are expected to arise from the randomization process, deviations from the planned intervention, poor measurement of outcome, missing outcome data, and selective reporting issues. The overall risk for each of these issues will be graded as low, medium, and high. In case of an agreement during this process, we will address it through discussion.

#### 2.3.4 Measures of treatment effect

We will use relative risk and 95% confidence intervals to analyze dichotomous data and estimate the effect of treatment. Also, we intend to utilize the mean differences or standardized mean differences with 95% confidence level to analyze continuous data.

#### 2.3.5 Assessment of heterogeneity

We will measure heterogeneity with the I2 statistic before pooling any outcome. In case of a significant heterogeneity (I2 > 50%), we will use the model of random-effect for analysis. Where there is no heterogeneity, we will adopt the model of fixed-effect.

#### 2.3.6 Assessment of publication biases

Where 10 or more studies are included, we intend to evaluate funnel plot asymmetry to report biases and utilize the Egger test to establish the small-study effects.

#### 2.3.7 Sensitivity analysis

We will perform a sensitivity analysis to determine the robustness and reliability of our results by excluding studies at high risk of bias.

## 3 Discussion

This present study will be conducted to assess the clinical therapeutic impacts of *H pylori* eradication to treat patients experiencing T2DM. We consider that there is no systematic review or meta-analysis that has investigated this concern. Therefore, we consider that this study is meaningful to evaluate the clinical therapeutic effects of *H pylori* eradication for patients with T2DM.

Our study will search related studies without any language restrictions. Also, we will fully consider all potential studies concerning the clinical therapeutic impacts of *H pylori* eradication in patients with T2DM. We anticipate drawing conclusion that can present a solid data for the future research protocols. We will also provide an up-to-date summary of the current evidence on the clinical therapeutic impacts of *H pylori* eradication for patients experiencing T2DM. We believe that these findings can help bring evidence for clinicians.

## Author contributions

**Conceptualization:** Zhi-Guo Shi, Li-Hui Chen.

**Data curation:** Zhi-Guo Shi, Li-Hui Chen.

**Formal analysis:** Zhi-Guo Shi.

**Funding acquisition:** Li-Hui Chen.

**Investigation:** Zhi-Guo Shi.

**Methodology:** Zhi-Guo Shi, Li-Hui Chen.

**Resources:** Zhi-Guo Shi, Li-Hui Chen.

**Software:** Zhi-Guo Shi.

**Supervision:** Zhi-Guo Shi, Li-Hui Chen.

**Validation:** Zhi-Guo Shi.

**Visualization:** Zhi-Guo Shi, Li-Hui Chen.

**Writing – original draft:** Zhi-Guo Shi.

**Writing – review & editing:** Li-Hui Chen.
